# Surface and Interface Modulation of V_2_O_5_/Ni(OH)_2_ Nanomaterials for Enhanced Alkaline Water Splitting

**DOI:** 10.3390/molecules31010113

**Published:** 2025-12-29

**Authors:** Jia Feng, Yongren Yu, Yinxin Zhang, Haojie Sun, Xiaomei Wang, Shiwei Song, Yucai Li, Jian Wang, Depeng Zhao, Fang Hu

**Affiliations:** 1 School of New Energy, Shenyang Institute of Engineering, Shenyang 110136, China; 2 School of Materials Science and Engineering, Shenyang University of Technology, Shenyang 110870, China

**Keywords:** electrocatalyst, water splitting, electrochemical hydrogen production

## Abstract

To optimize the electrocatalytic reaction process through the synergistic effects of V and Ni, this study employed a two-step hydrothermal method to successfully construct a V_2_O_5_ composite structure grown on a Ni(OH)_2_ substrate (denoted V_2_O_5_/Ni(OH)_2_-2). Electrochemical evaluation revealed that this catalyst exhibits efficient bifunctional activity in 1.0 M KOH electrolyte. For the hydrogen evolution reaction (HER), it requires a mere 89.6 mV overpotential to achieve a current density of −10 mA cm^−2^. The catalyst also demonstrates excellent performance in the oxygen evolution reaction (OER), demanding only 198 mV overpotential to drive a current density of 10 mA cm^−2^, while maintaining low overpotential increases even at high current densities. Furthermore, it exhibits outstanding long-term stability during a 12 h continuous test. When assembled as a dual-electrode overall water splitting device, the system requires a voltage of only 2.82 V to drive a high current density of 100 mA cm^−2^, showcasing its significant potential for practical applications.

## 1. Introduction

The overreliance on fossil fuels has not only exacerbated the depletion of non-renewable energy resources but also accelerated environmental degradation, fueling intense interest in exploring and utilizing renewable energy [1,2,3,4,5,6]. Among various approaches, water splitting for hydrogen production and photovoltaic power generation provide environmentally friendly routes to generate clean energy and have become key research priorities [7,8]. Hydrogen, characterized by its high energy density and environmental friendliness, represents an excellent solution for addressing the energy crisis [9]. Currently, commercial hydrogen and oxygen evolution catalysts predominantly include Pt/C, IrO_2_, and RuO_2_ [10,11,12,13,14]. However, these materials are largely based on precious metals, whose prohibitively high costs severely limit their widespread application. Consequently, the research and design of catalysts possessing superior catalytic performance with low cost has emerged as a major focus [15,16,17,18,19,20].

Liu et al. [21] synthesized a strongly coupled heterostructure catalyst composed of NiCo-LDHs nano-octahedra using an oil-bath method. The bimetallic synergy within the resulting CuS@NiCo-LDHs heterostructure not only provides abundant active sites but also enhances electrical conductivity and accelerates electron transport. In another study, Wang et al. [22] designed a high-valent Zr-doped bimetallic sulfide (NF/Ni@Zr-NiCoS), which was grown directly on roughened nickel foam through electrodeposition followed by a two-step hydrothermal process. This catalyst required overpotentials of only 216 mV and 275 mV to achieve current densities of 10 mA cm^−2^ and 100 mA cm^−2^, respectively. Furthermore, it displayed robust stability and durability, operating steadily for 100 h at 200 mA cm^−2^. Similarly, Wang et al. [23] synthesized Fe-doped and carboxylate-modified bimetallic phosphide (NCFCP@NF) electrocatalysts via in-situ growth on nickel foam. This approach provided a greater number of active sites, significantly improving catalytic performance. For the hydrogen evolution reaction (HER), it required overpotentials of just 25 mV and 283 mV to reach current densities of 10 mA cm^−2^ and 3000 mA cm^−2^, respectively. For the oxygen evolution reaction (OER), the overpotentials were only 163 mV and 329 mV to deliver the same current densities. Notably, a two-electrode setup for overall water splitting achieved a current density of 10 mA cm^−2^ at a cell voltage of only 1.42 V. The research results reported above have made some progress, but the study of high-performance catalysts is still a hot topic. On this basis, a bifunctional V_2_O_5_/Ni (OH)_2_ electrocatalyst was successfully prepared by the hydrothermal method using nickel foam as the nickel source and substrate. The chemical composition, morphology, and structure of the obtained materials were analyzed in detail by a series of characterization techniques. On this basis, the electrocatalytic performance of the sample in hydrogen and oxygen evolution reactions was studied by a three-electrode system. In HER, the overpotential of V_2_O_5_/Ni (OH)_2_-2 material is 89.6 mV at a current density of 10 mA cm^−2^, the Tafel slope value is 136.1 mV dec^−1^, and the Cdl value is 0.308 mF cm^−2^. In OER, at a current density of 10 mA cm^−2^, the overpotential of V_2_O_5_/Ni (OH)_2_-2 material is 198 mV, the Tafel slope value is 113.2 mV dec^−1^, and the Cdl value is 0.43 mF cm^−2^. The catalytic performance is significantly better than other materials (Table 1).

## 2. Results and Discussion

**Figure 1:**
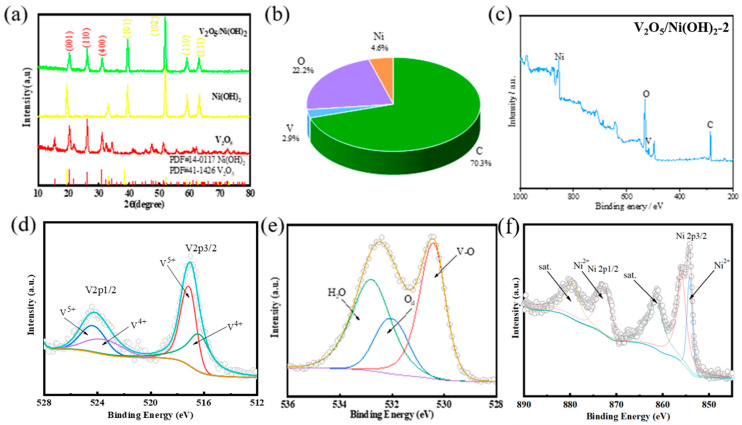
(**a**) XRD patterns of V_2_O_5_/Ni(OH)_2_ samples (**b**) percentages of each element (**c**) XPS full spectra, XPS spectra of Ni(OH)_2_ samples (**c**) XPS full spectra (**d**) V 2p (**e**) O 1s (**f**) Ni 2p.

Because the preparation of the material is directly grown on nickel foam, it is necessary to eliminate the interference of Ni on the derivative peak of V_2_O_5_/Ni (OH)_2_ material when detecting XRD. Therefore, V_2_O_5_/Ni (OH)_2_ powders grown on nickel foam were obtained by scraping or ultrasonic vibration. The XRD image of the V_2_O_5_/Ni (OH)_2_ material detected is shown in Figure 1a below. The red line represents the pure V_2_O_5_ material, and there is no excess impurity peak. The diffraction peaks at 20.3°, 26° and 31° correspond to the (001), (110) and (400) planes of the V_2_O_5_ phase (JCPDS number 41-1426), respectively. The other derivative peaks are the (101), (102), (110), and (111) crystal planes belonging to the Ni(OH)_2_ phase (JCPDS number 14-0117), and the corresponding 2θ are 38.5°, 52.16°, 59.1° and 62.8°, respectively. It can also be proved by XRD images that the V_2_O_5_ and Ni(OH)_2_ phases in the material V_2_O_5_/Ni (OH)_2_ coexist in the material, which indicates that the composite material V_2_O_5_/Ni (OH)_2_ is correctly synthesized (Table 2 and Table 3).

X-ray photoelectron spectroscopy (XPS) was employed to investigate the elemental composition and chemical valence states of the synthesized V_2_O_5_/Ni(OH)_2_ and its constituent materials, confirming consistency with the experimental design. The survey spectrum (Figure 1c) clearly indicated the presence of the primary elements: Ni, O, C, and V. The quantitative analysis cited from Figure 1b listed contents of 4.6%, 22.2%, 70.3%, and 2.9%, respectively, which appears to have a numerical discrepancy. The high-resolution V 2p spectrum (Figure 1d) revealed significant changes in the vanadium oxidation state. Due to spin-orbit coupling, this spectrum was deconvoluted into V 2p_3_/_2_ and V 2p_1_/_2_ components. The peaks centered at 517.2 eV and 524.31 eV, separated by 7.1 eV, are characteristic of V^5+^ (attributed to vanadium pentoxide). Concurrently, distinct peaks corresponding to the V^4+^ valence state were identified at binding energies of 516.2 eV and 523.4 eV. This observed shift and the coexistence of multiple vanadium oxidation states strongly indicate the successful preparation of the V_2_O_5_/Ni(OH)_2_ composite. Following this, a careful observation of the O 1s core-level XPS spectrum (Figure 1e) shows that it comprises three main peaks. Among them, two peaks are located at 530.5 eV and 532.5 eV, respectively, belonging to O and OH of V_2_O_5_/Ni(OH)_2_@NF catalyst, and the other peak is located at 533.1 eV, which is adsorbed water [27,28]. Finally, as shown in Figure 1f, in the Ni 2p XPS spectrum, the peaks with binding energies of 855.3 and 873.0 eV are attributed to Ni 2p_3/2_ and Ni 2p_1/2_, respectively, and the spin energy separation is 17.7 eV, which is the characteristic feature of Ni^2+^ in Ni(OH)_2_. The two jitter satellite peaks of Ni 2p_3/2_ and Ni 2p_1/2_ are located at about 861.4 eV and 879.4 eV, respectively [26,27,29,30,31].

**Figure 2:**
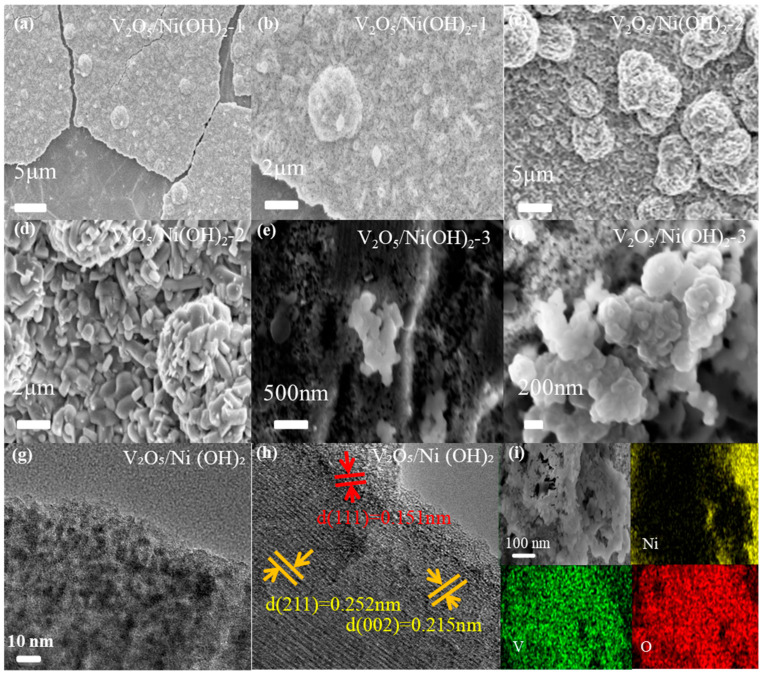
SEM images of (**a**,**b**) V_2_O_5_/Ni(OH)_2_-1 (**c**,**d**) V_2_O_5_/Ni(OH)_2_-2 (**e**,**f**) V_2_O_5_/Ni(OH)_2_-3 (**g**,**h**) HRTEM morphology of the as-prepared materials (**i**) Element mapping diagram of V_2_O_5_/Ni(OH)_2_ sample.

Figure 2 illustrates the surface morphological features of the as-prepared catalysts, as characterized by SEM and TEM. Figure 2a, the SEM image of V_2_O_5_/Ni(OH)_2_-1, reveals that nanoclusters were successfully grown on the nanosheet substrate. However, these nanoclusters are sparsely distributed, indicating fewer available reactive active sites. A magnified view of these clusters is presented in Figure 2b. For the V_2_O_5_/Ni(OH)_2_-2 sample (Figure 2c), it is evident that with an increased V content, the density of cluster structures on the nanosheet surface significantly increases. This enhancement in active sites leads to improved electrochemical performance. The magnified image (Figure 2d) clearly indicates that the clusters are formed by the aggregation of small nanoblocks. These structures are interwoven and stacked, creating an abundance of irregular pores. This nanostructure further constitutes a micron-scale macroporous framework, which greatly facilitates mass transport and electrolyte penetration. Such a hierarchical pore structure and 3D interconnected network offer significant advantages for electrocatalysis. Figure 2e,f displays the morphology of V_2_O_5_/Ni(OH)_2_-3. As the V content is further increased, both the nanosheet and cluster structures diminish. The high-magnification SEM image (Figure 2f) reveals that this irregular structure is formed by the agglomeration of numerous nanospheres, with fine nanoscale particles distributed on the surface, resulting in a significantly smaller overall size. For a detailed structural analysis, high-resolution transmission electron microscopy (HRTEM) images are provided in Figure 2g,h. The V_2_O_5_/Ni(OH)_2_-2 sample, which exhibited the optimal morphology, was selected for HRTEM characterization. The observed lattice fringes of 0.215 nm and 0.252 nm correspond to the (002) and (211) crystal planes of V_2_O_5_, respectively. Another distinct lattice spacing of 0.151 nm, observed in the selected area, is in good agreement with the (111) plane of Ni(OH)_2_. These TEM analysis results are highly consistent with the XRD data, further verifying the successful formation of the V_2_O_5_/Ni(OH)_2_ composite. As shown in Figure 2i, the elemental mapping images of the material indicate that V and O elements are uniformly distributed throughout the central region, whereas Ni is primarily enriched at the edges. This distribution further confirms the successful synthesis of the composite material.

**Figure 3:**
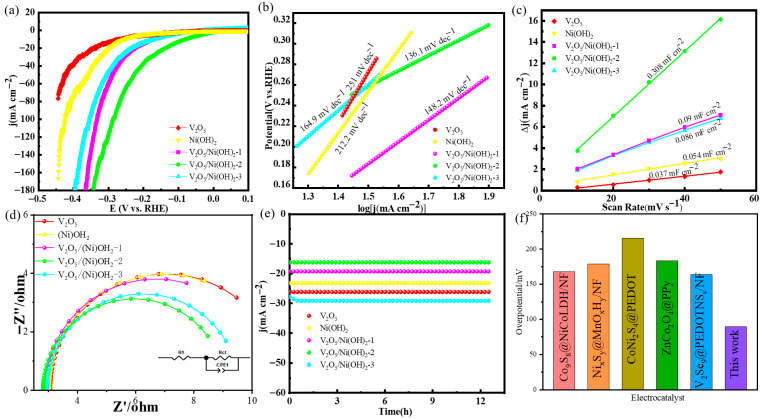
HER performance: (**a**) LSV curves; (**b**) Tafel plots; (**c**) double-layer capacitance measurement linear fitting; (**d**) Nyquist plots; (**e**) chronoamperometric stability tests. (**f**) Comparison of overpotential for HER.

The hydrogen evolution performance of electrocatalysts was discussed. A three-electrode system was assembled by using synthetic materials and experimental devices such as an electrolytic cell, carbon sheet, platinum sheet, and silver chloride to study the catalytic performance of HER and OER of several synthetic materials. The scan rate for LSV testing is 5 mV s^−1^, which balances test efficiency with data accuracy and avoids capacitive current interference. The LSV curves of the five samples are shown in Figure 3a. It can be seen from the figure that the overpotential of the V_2_O_5_/Ni(OH)_2_-2 material is 89.6 mV at a current density of 10 mA cm^−2^, which is smaller than that of the single V_2_O_5_ (251 mV) and Ni(OH)_2_ (214.8 mV) materials. This also shows that the increase in HER performance is due to the synergistic effect between V_2_O_5_ and Ni(OH)_2_. At the same time, the overpotential of V_2_O_5_/Ni(OH)_2_-2 sample is much smaller than that of V_2_O_5_/Ni(OH)_2_-1 (193.9 mV) and V_2_O_5_/Ni(OH)_2_-3 (168.2 mV). In order to further explore the reaction kinetics of the material, the Tafel slope is calculated by linearly fitting the LSV curve to a polarization curve, as shown in Figure 3b. The Tafel slope of V_2_O_5_/Ni(OH)_2_-2 is 136.1 mV dec^−1^, which is obviously lower than that of the two pure V_2_O_5_ and Ni (OH)_2_ materials. The Tafel values are 268 mV dec^−1^ and 212 mV dec^−1^, respectively. When compared with other materials, V_2_O_5_/Ni(OH)_2_-2 is also significantly lower than V_2_O_5_/Ni(OH)_2_-1 (148.2 mV dec^−1^) and V_2_O_5_/Ni(OH)_2_-3 (164.9 mV dec^−1^). Therefore, it can be concluded that a certain proportion of V and Ni elements can enhance the rapid occurrence of the HER reaction process. Compared with the pure sample, it can be concluded that the structure of the composite structure can greatly improve the kinetics of the HER reaction. According to the Volmer–Heyrovsky mechanism, the smaller Tafel value has a faster reaction rate, which is completely consistent with the above experiments, so V_2_O_5_/Ni(OH)_2_-2 is the best sample. In the process of electrochemical reaction, most of the chemical reactions of active substances occur on the surface, and the specific surface area can affect the rate and efficiency of the reaction to a certain extent. Therefore, the larger comparative area can provide more reaction sites and increase the contact area between the reaction substances so as to achieve the effect of improving the reaction. This is the principle of electrochemically active surface area (ECSA). However, there is a direct relationship between ECSA and Cdl, and then ECSA is obtained by measuring Cdl, and finally the kinetics of electrochemical reaction is analyzed. As shown in Figure 3c, the Cdl value of V_2_O_5_/Ni(OH)_2_-2 material is 0.308 mF cm^−2^, which is much larger than that of the other four materials, and the gap between the materials is significant. The Cdl values of V_2_O_5_/Ni(OH)_2_-1, V_2_O_5_/Ni(OH)_2_-3, V_2_O_5_ and Ni(OH)_2_ are 0.09 mF cm^−2^, 0.086 mF cm^−2^, 0.054 mF cm^−2^ and 0.037 mF cm^−2^, respectively. It shows that the specific surface area of V_2_O_5_/Ni(OH)_2_-2 material is the largest, the number of active sites is the largest, and the reaction rate is the fastest. It is concluded that the amount of Ni element added will change the morphology of these materials and then change the specific surface area of the composite material, indirectly changing the electrocatalytic performance of the material [30,31]. Through the experiment of electrochemical impedance, the charge transfer performance of the electrode or electrolyte and the kinetics of the reaction process are explored. As shown in Figure 3d, during the HER EIS measurements, V_2_O_5_/Ni(OH)_2_-2 exhibits the smallest semicircle diameter, indicating the lowest Rct of 9.7 Ω and Rs of 2.584 Ω. In contrast, V_2_O_5_/Ni(OH)_2_-1 and V_2_O_5_/Ni(OH)_2_-3 display significantly increased Rct values of 13.1 Ω and 17.3 Ω, respectively, due to insufficient active sites, which substantially hinder reaction kinetics. The stability of the material is another criterion for the speed of the electrochemical reaction to a certain extent. Because good stability represents that the material can maintain excellent reactivity for as long as possible in a certain period of time at −1.12 V, it can be found from Figure 3e that the overpotential of these materials does not change after 24 h at current densities of 26, 24, 20, 16 and 28 mA cm^−2^ (at −0.24, −0.25, −0.14, −0.17, and −0.23 V). This indicates that the synthesized materials V_2_O_5_/Ni(OH)_2_-1, V_2_O_5_/Ni(OH)_2_-2, V_2_O_5_/Ni(OH)_2_-3, V_2_O_5_ and Ni(OH)_2_ can remain stable in the HER reaction. Electrocatalytic performance of V_2_O_5_/Ni(OH)_2_-2 nanowires is comparable with other reported catalysts, as presented in Figure 3f. The as-prepared catalysts possess more excellent performance than others [29,32,33,34,35].

**Figure 4:**
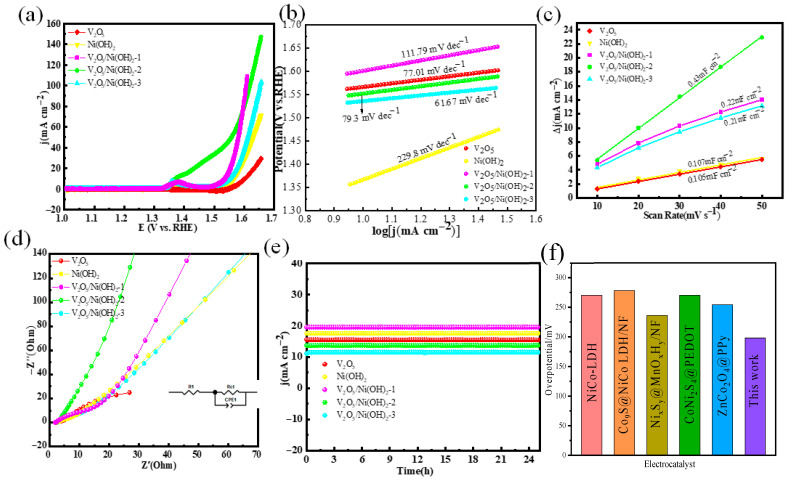
OER performance: (**a**) LSV curves; (**b**) Tafel plots; (**c**) double-layer capacitance measurement linear fitting; (**d**) Nyquist plots; (**e**) chronoamperometric stability tests. (**f**) Comparison of overpotential for OER.

To assess whether the oxygen evolution reaction (OER) performance aligns with the previously observed hydrogen evolution reaction (HER) activity, the electrocatalytic properties of V_2_O_5_/Ni(OH)_2_, V_2_O_5_/Ni(OH)_2_-1, V_2_O_5_/Ni(OH)_2_-2, V_2_O_5_/Ni(OH)_2_-3, and their single-component counterparts were systematically evaluated under identical experimental conditions. As shown in the OER polarization curves (Figure 4a), V_2_O_5_/Ni(OH)_2_-2 exhibited the highest catalytic activity, requiring an overpotential of only 198 mV to achieve a current density of 10 mA cm^−2^—significantly lower than those of V_2_O_5_/Ni(OH)_2_-1 (304 mV), V_2_O_5_/Ni(OH)_2_-3 (326 mV), pristine V_2_O_5_ (352 mV), and Ni(OH)_2_ (331 mV). These results demonstrate that the heterostructured composite enhances OER activity; however, excessive Ni content beyond the optimal ratio in V_2_O_5_/Ni(OH)_2_-2 leads to performance degradation, underscoring the critical importance of compositional balance in maximizing accessible active sites. Kinetic analysis via Tafel plots (Figure 4b) further corroborates this trend: V_2_O_5_/Ni(OH)_2_-2 displays the lowest Tafel slope of 79.3 mV dec^−1^, markedly outperforming Ni(OH)_2_ (229.8 mV dec^−1^) and V_2_O_5_/Ni(OH)_2_-3 (111.79 mV dec^−1^), thereby confirming its superior charge transfer kinetics during the OER process. To quantify the electrochemically active surface area (ECSA), double-layer capacitance (C_dl_) measurements were conducted (Figure 4c). V_2_O_5_/Ni(OH)_2_-2 exhibits the highest C_dl_ value of 0.43 mF cm^−2^, substantially exceeding those of all other samples, which indicates a larger specific surface area and a greater density of exposed catalytically active sites. The Nyquist plot for OER (Figure 4d) reveals that the solution resistance (Rs = 2.51 Ω) is nearly identical for all samples, while V_2_O_5_/Ni(OH)_2_-2 maintains the lowest Rct value of 9.8 Ω. These results suggest that both excess and deficient vanadium (V) content compromise the structural integrity of the catalyst, thereby increasing the charge transfer resistance. This analysis provides insights into the critical role of optimal V composition in enhancing the catalytic performance by minimizing Rct, which is crucial for improving the efficiency of water electrolysis reactions. Long-term stability tests were conducted at 0.54 V; Figure 4e confirms that all synthesized catalysts maintain robust activity over a 24 h chronoamperometric measurement, with negligible performance decay. Notably, as summarized in Figure 4f, the OER performance of the as-prepared V_2_O_5_/Ni(OH)_2_-2 nanowires is highly competitive—and in several metrics superior—to those of recently reported state-of-the-art electrocatalysts [28,32,36,37,38,39,40].

**Figure 5:**
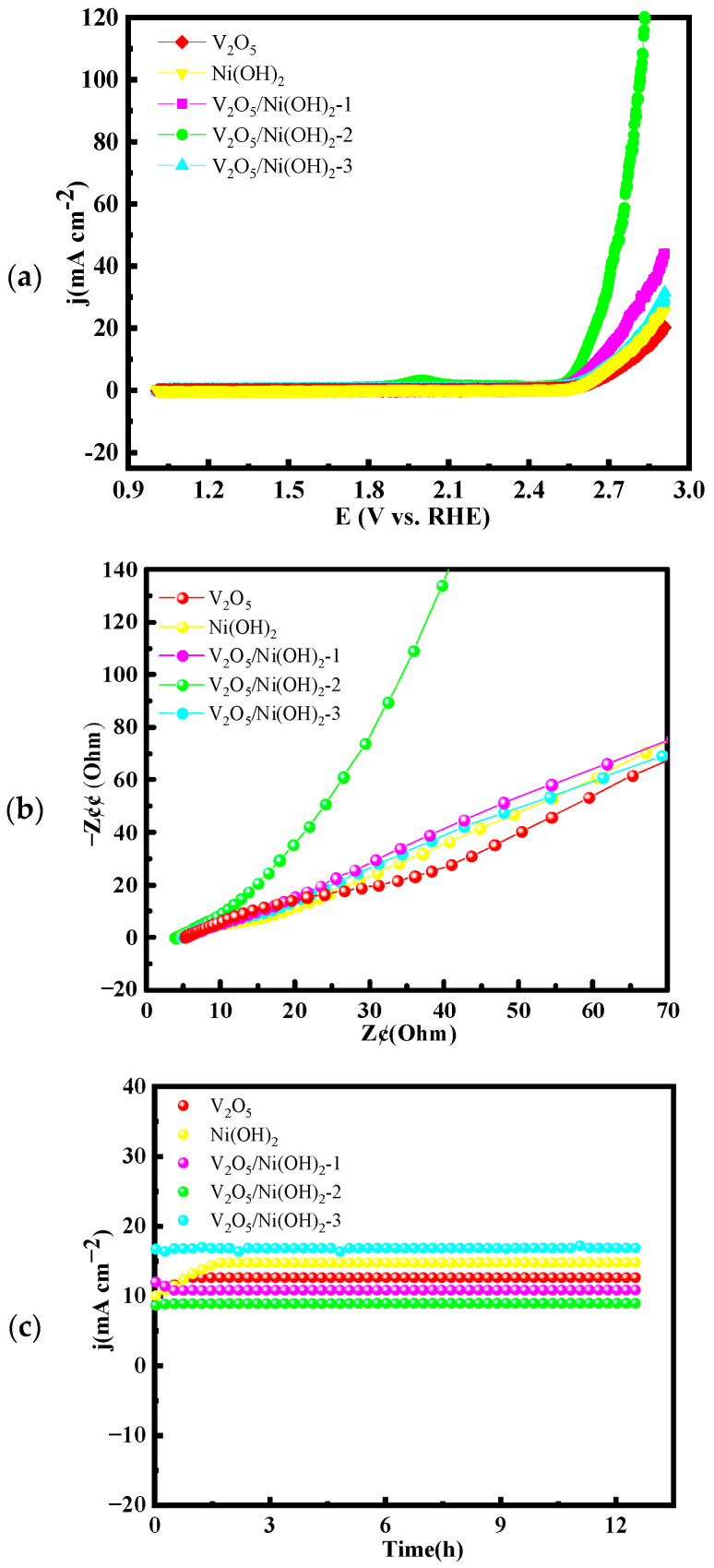
Overall water splitting performance of: (**a**) LSV curves; (**b**) Nyquist plots; (**c**) chronoamperometric stability tests.

A simple two-electrode electrolytic cell, with both electrodes loaded with the as-prepared samples, was employed to construct a full water-splitting system for evaluating the overall electrocatalytic performance of the synthesized materials as bifunctional catalysts. As shown in Figure 5, the electrochemical characterization results of the overall water-splitting system are presented. Figure 5a displays the polarization curves of all synthesized materials. Among them, V_2_O_5_/Ni(OH)_2_-2 exhibits the best performance, requiring only 1.59 V to achieve a current density of 10 mA cm^−2^. In comparison, V_2_O_5_, Ni(OH)_2_, V_2_O_5_/Ni(OH)_2_-1, and V_2_O_5_/Ni(OH)_2_-3 require higher cell voltages of 1.78 V, 1.80 V, 1.72 V, and 1.75 V, respectively, at the same current density. Moreover, V_2_O_5_/Ni(OH)_2_-2 demonstrates exceptional activity even at high current densities, needing only 2.82 V to reach 100 mA cm^−2^, further confirming its superior catalytic efficiency. Figure 5b presents the electrochemical impedance spectroscopy (EIS) data. V_2_O_5_/Ni(OH)_2_-2 shows a steeper linear slope in the low-frequency region compared to V_2_O_5_, Ni(OH)_2_, V_2_O_5_/Ni(OH)_2_-1, and V_2_O_5_/Ni(OH)_2_-3, indicating the lowest ion diffusion resistance and the fastest reaction kinetics.

The long-term stability of the catalysts during overall water splitting is illustrated in 1.6 V (Figure 5c). After 12 h of continuous electrolysis, V_2_O_5_/Ni(OH)_2_-2 maintains excellent stability with negligible fluctuation in current density. In contrast, Ni(OH)_2_ and V_2_O_5_/Ni(OH)_2_-3 exhibit noticeable current variations, underscoring the outstanding durability and overall electrocatalytic performance of V_2_O_5_/Ni(OH)_2_-2 for practical applications.

## 3. Experimental

### 3.1. Materials

Ammonium metavanadate (NH_4_VO_3_), Sodium hydroxide (NaOH), Sodium hydroxide ((NH_4_)_2_S_2_O_8_), nickel nitrate hexahydrate [Ni(NO_3_)_2_·6H_2_O, 99%], ammonium fluoride (NH_4_F, 96%), urea (H_2_NCONH_2_, 99.5%), sodium hypophosphite potassium hydroxide (KOH), absolute ethanol (C_2_H_6_O, 99.5%), hydrochloric acid (HCl), and nickel foam were all of analytical grade and purchased from Sigma-Aldrich (St. Louis, MO, USA). These chemicals were used without further purification.

### 3.2. Materials Synthesis

#### Preparation of V_2_O_5_/Ni(OH)_2_ on NF

This chapter is to synthesize composite materials by using a simple solvothermal experimental method. First, the foam nickel required for the experiment needs to be prepared in advance; that is, a 3 × 3 cm^2^ large square is cut from the complete foam nickel and then immersed in 1.5 mol/L hydrochloric acid to clean the oil and impurities present on the surface. Finally, the soaked materials were repeatedly washed with deionized water and ethanol and dried for later use. 1.54 mmol of ammonium metavanadate, 9 mmol of sodium hydroxide, and 25 mmol of urea were dissolved in 15 mL of distillation, and then the stirred solution was clarified by ultrasonic value solution. Then, 1.1 g of ammonium persulfate solution was added for stirring at room temperature. Finally, the solution was transferred into the reactor, and the prepared nickel foam was added. React for 8 h at a temperature of 170 °C. The prepared materials were dried after the reaction. Place the initially prepared sample into 60 mL of deionized water dissolved with 1 mmol Ni(NO_3_)_2_, 8 mmol urea, and 15 mmol NH_4_F, and carry out a secondary hydrothermal treatment for 4 h at 120 °C, with the ratio of Ni to V being 1:1, 1:2, and 1:3.

### 3.3. Materials Characterization

The crystal structure of the samples was characterized by X-ray diffraction (XRD, Shimadzu-7000, Cu Kα, Shimadzu, Kyoto, Japan). X-ray photoelectron spectroscopy (XPS, ESCALAB 250 with an Al Kα source, Thermo Fisher Scientific, Waltham, MA, USA) was used to analyze surface chemical states and provide qualitative and quantitative elemental information. The morphology of the samples was examined by scanning electron microscopy (SEM, Gemini 300-71-31, Carl Zeiss, Oberkochen, Germany) and transmission electron microscopy (TEM, JEM-2100 PLUS, JEOL, Tokyo, Japan). Results showed well-defined microstructures.

### 3.4. Electrochemical Measurements

All samples were characterized using a CHI 760E electrochemical workstation (Shanghai Chenhua Instrument Co., Ltd., Shanghai, China). The as-prepared electrocatalytic samples served as the working electrode, while Hg/HgO was used as the reference electrode. A graphite carbon rod and platinum sheet acted as the counter electrodes for the hydrogen evolution reaction (HER) and oxygen evolution reaction (OER), respectively. The electrolytes consisted of a 1.0 M KOH solution. Linear sweep voltammetry (LSV) curves with 90% IR compensation were corrected using the Nernst equation (E_RHE_ = E_Hg/HgO_ + 0.098 + 0.0591 × pH). The OER overpotential was calculated based on the thermodynamic reference (E vs. RHE = 1.23 V). The electrolytic cell incorporates two electrode clips that hold catalyst samples of identical geometric area, serving as the positive and negative electrodes, with a 50 mL 1.0 M KOH solution as the electrolyte for the test.

## 4. Conclusions

A controlled two-step hydrothermal method was employed to successfully design and synthesize a V_2_O_5_/Ni(OH)_2_ composite on nickel foam substrates. Among the series, the V_2_O_5_/Ni(OH)_2_-2 sample with the optimized V/Ni molar ratio exhibited the most superior bifunctional electrocatalytic activity. In 1.0 M KOH electrolyte, this composite demonstrated exceptional HER performance, requiring only 89.6 mV overpotential to achieve a current density of −10 mA cm^−2^, markedly lower than those of pristine V_2_O_5_ (251 mV) and Ni(OH)_2_ (214.8 mV). Simultaneously, it displayed outstanding OER activity with a mere 198 mV overpotential at 10 mA cm^−2^, significantly outperforming V_2_O_5_ (352 mV) and Ni(OH)_2_ (331 mV). When configured as both anode and cathode in a two-electrode overall water splitting system, the V_2_O_5_/Ni(OH)_2_-2 electrode required only 1.59 V cell voltage to deliver 10 mA cm^−2^, maintaining robust stability over 12 h of continuous operation. Performance enhancement is primarily attributed to strong synergistic effects between V_2_O_5_ and Ni(OH)_2_, which generated a unique 3D hierarchical porous morphology. This architecture substantially increased the electrochemical active surface area (ECSA), optimized electronic structure, and reduced charge transfer resistance (Rct). This study provides an effective strategy for designing efficient, stable, and cost-effective vanadium-based bifunctional water-splitting catalysts.

## Figures and Tables

**Table 1:** The electrocatalytic performance of the samples.

Materials	Overpotential (mV)	Electrolyte	Ref.
NiCoP/NF	32 (−10 mA cm^−2^)	1.0 M KOH	[24]
	280 (10 mA cm^−2^)		
CoS_2_ HNSs	-	1.0 M KOH	[25]
	290 (10 mA cm^−2^)		
NiCo_2_O_4_	110 (−10 mA cm^−2^)	1.0 M NaOH	[26]
	290 (10 mA cm^−2^)		
V_2_O_5_/Ni(OH)_2_-2	89 (−10 mA cm^−2^)	1.0 M KOH	This work
	198 (10 mA cm^−2^)		

**Table 2:** The composition ratio of the prepared electrode materials (first hydrothermal).

Materials	NH_4_VO_3_	Urea (First Hydrothermal)	Na(OH)	(NH_4_)_2_S_2_O_8_
V_2_O_5_/Ni(OH)_2_-1	1.54 mmol	25 mmol	9 mmol	1.1 g
V_2_O_5_/Ni(OH)_2_-2	1.54 mmol	25 mmol	9 mmol	1.1 g
V_2_O_5_/Ni(OH)_2_-3	1.54 mmol	25 mmol	9 mmol	1.1 g

**Table 3:** The composition ratio of the prepared electrode materials (secondary hydrothermal).

Materials	NiNO_3_	Urea (First Hydrothermal)	NH_4_F
V_2_O_5_/Ni(OH)_2_-1	1 mmol	8 mmol	15 mmol
V_2_O_5_/Ni(OH)_2_-2	2 mmol	8 mmol	15 mmol
V_2_O_5_/Ni(OH)_2_-3	3 mmol	8 mmol	15 mmol

## Data Availability

The original contributions presented in this study are included in the article. Further inquiries can be directed to the corresponding author(s).
